# Further characterization of infection mechanisms and transmission dynamics of rustrela virus in rodent in vivo models

**DOI:** 10.1099/jgv.0.002266

**Published:** 2026-05-19

**Authors:** Sophie-Celine Weinert, Lorenz Ulrich, Lukas M. Michaely, Vinícius Pinho dos Reis, Claudia Wylezich, Dirk Höper, Martin Beer, Angele Breithaupt, Dennis Rubbenstroth, Kore Schlottau

**Affiliations:** 1 Institute of Diagnostic Virology, Friedrich-Loeffler-Institut, Greifswald-Insel Riems, Germany; 2 Department of Experimental Animal Facilities and Biorisk Management, Friedrich-Loeffler-Institut, Greifswald-Insel Riems, Germany

**Keywords:** dead-end host, experimental infection, *Matonaviridae*, reservoir host, rustrela virus, transmission

## Abstract

Rustrela virus (RusV) is a newly discovered member of the genus *Rubivirus*. RusV causes a fatal, non-suppurative meningoencephalomyelitis in a variety of mammals. Various aspects of RusV biology remain poorly understood, e.g. the excretion of infectious virus and its transmission. To address these questions, we performed *in vivo* experiments including putative reservoir (wood mice) as well as potential dead-end hosts (Lewis rats). To investigate the natural route of RusV infection and transmission, subgroups of wood mice and Lewis rats were either inoculated intranasally or orally. Successful infection was only observed in animals inoculated via the intranasal route, leading to the assumption that the virus could enter via olfactory neurons and subsequently spread throughout the central nervous system. To gain an initial understanding of the mechanisms of viral transmission, we co-housed RusV-inoculated wood mice with non-infected sentinels. In addition, wood mice or Lewis rats were placed in cages previously occupied by infected wood mice as donor animals for indirect transmission via the environment. Successful RusV transmission was observed solely in co-housed sentinels. These findings suggest that RusV transmission requires direct contact with bodily fluids, such as saliva or nasal secretions or at least a higher infective dose. In contrast, indirect exposure to a presumably contaminated cage does not seem to be sufficient for transmission between reservoir animals or reservoir and dead-end hosts.

## Data Summary

The authors confirm that all supporting data and protocols have been provided within the article or through supplementary data files. Additional datasets not shown in the manuscript can be obtained from the authors upon request.

## Introduction

Until recently, the human-pathogenic rubella virus (RuV) (*Rubivirus rubellae*) was the sole member of the genus *Rubivirus* and the only virus classified within the *Matonaviridae* family of single-stranded, positive-sense RNA viruses [1,2]. In 2020, two novel rubiviruses were discovered: rustrela virus (RusV) (*Rubivirus strelense*) and ruhugu virus (*Rubivirus ruteetense*), which were both identified as close relatives of RuV by metagenomic sequencing [3]. Subsequently, other more divergent matonaviruses were identified in fish and reptiles [1,3,7]. Thereupon, RusV was identified as the causative agent of fatal nonsuppurative meningoencephalitis in a broad range of mammalian species housed in a zoological garden in northeastern Germany [3,8, 9]. Retrospective analysis of nonsuppurative encephalitides of uncertain aetiology led to the detection of RusV in archived brain tissue of lions (*Panthera leo*) that had died in the 1970s and 1980s in zoological gardens in Germany [10,11]. Moreover, previous studies identified RusV as the causative agent of ‘staggering disease’, a rare but severe neurologic disease affecting domestic cats (*Felis catus*), particularly in Sweden but also in Austria and Germany [12,14]. Notably, an occurrence outside of Europe and an extension of the host spectrum of RusV was reported with the detection of RusV in a wild mountain lion (*Puma concolor*) from CO, USA, which presented with severe neurological symptoms consistent with those observed in cases of staggering disease in domestic cats [15].

RusV exhibits a strong neurotropism, as evidenced by the detection of viral RNA predominantly within the central nervous system (CNS) of naturally as well as experimentally infected mammals, with remarkably low levels of viral RNA detected in extraneural tissues, such as the liver or small intestine [3,9, 10, 12, 14, 16, 17]. Neurons are the predominant target of RusV, and to a lesser extent, astrocytes and microglial cells, as evidenced by RNA *in situ* hybridization (RNA-ISH) and immunohistochemistry [3,8, 10, 12, 14, 16, 17].

As reported in previous studies, RusV RNA has been detected in wild rodents of the genus *Apodemus*, which includes the yellow-necked field mouse (*Apodemus flavicollis*) and the wood mouse (*Apodemus sylvaticus*). Interestingly, no histopathological lesions were detected in the brain tissue of these rodents, suggesting that they may act as a reservoir for RusV [3,8, 14, 18]. Moreover, the detection of viral RNA in tissues associated with viral shedding, such as in the parotid gland or urinary bladder of experimentally infected wood mice, provides further support for this hypothesis [16].

Several aspects of RusV’s biology remain poorly understood. These aspects include the natural route of infection, viral shedding and the transmission between reservoir hosts and to dead-end hosts. Initial experimental infection studies performed by Klein *et al.* provided the first RusV infection model by successfully infecting wood mice and Lewis rats with brain homogenates derived from RusV-infected animals via different inoculation routes. In addition to intracerebral injection, only a combined intranasal (i.n.) and peroral (p.o.) inoculation, but not combined intramuscular and subcutaneous inoculation, led to successful infection, suggesting that natural infection may occur via the mucosal rather than the parenteral route [16]. Additionally, shedding of viral RNA was predominantly detected in oral swabs from wood mice, which emphasizes their potential role as a reservoir host for RusV [16].

In our study, we performed *in vivo* experiments to further investigate the natural RusV route of infection in wood mice and Lewis rats as well as to demonstrate intra- or interspecies transmission from wood mice to wood mice or Lewis rats, respectively. To this day, a cell culture-based *in vitro* model for RusV cultivation and propagation has not been established. Therefore, neuronal tissue samples from a previous trial [16] were used as inoculum. In the first experiment (Exp. 1), subgroups of wood mice and Lewis rats were inoculated either by i.n. or p.o. route to investigate the natural RusV route of infection. In the second experiment (Exp. 2), wood mice were exclusively inoculated i.n., and subgroups were euthanized at early timepoints to characterize viral kinetics. In the third experiment (Exp. 3), i.n. and p.o. inoculated wood mice were co-housed with uninfected sentinels of the same species. In addition, further uninfected wood mice and Lewis rats were exposed to potentially contaminated bedding material of the inoculated wood mice to test for indirect transmission efficacy.

## Methods

### Animal husbandry and biosafety

Animal welfare was monitored daily. Animals were fed on rodent pellets and water *ad libitum*. All animal and laboratory work involving infectious viruses was performed in biosafety level 2 laboratories at the Friedrich-Loeffler-Institut.

### Preparation of RusV inoculum (i.e. master inoculum)

Until today, a cell culture-based *in vitro* model for RusV cultivation and propagation has not been established. Hence, neuronal tissue samples from a previous trial [16], in which wood mice have been inoculated with virus originating from an infected cat from Sweden (GenBank accession number: ON641046.1; cat/SWE/SWE_15/2021; RusV clade 2A) [14], were used as a master inoculum.

Virus inoculum was prepared as follows: RusV-positive homogenized tissue samples (cerebrum, cerebellum and spinal cord) were thawed and thoroughly mixed. For the preparation of the master inoculum, supernatants from these samples were then pooled. Finally, 6 ml of sterile PBS was added to 34 ml of the pooled supernatants. Aliquots were stored at −80 °C until further use. The cycle of quantification (Cq) value of the inoculum was determined by RusV-specific reverse transcription-quantitative PCR (qPCR) (Cq 25.7).

### Next-generation sequencing

Prior to the *in vivo* experiments, an aliquot of the prepared RusV inoculum was subjected to metagenomic sequencing to identify potential alterations in the sequence upon animal passage. Sequencing libraries were generated according to Pfaff *et al*. [8] and sequenced on an Ion Torrent S5XL instrument using Ion 530 chips and chemistry for 400 bp reads (Thermo Fisher Scientific, Darmstadt, Germany). For sequence analysis of the RusV genome, the Genome Sequencer software suite (version 2.6; Roche, Mannheim, Germany) was used to perform mapping analysis against the reference sequence (GenBank accession number: ON641046.1). The datasets obtained were used to further test for the presence of any additional pathogens via the RIEMS pipeline [19].

No evidence of other viral or bacterial pathogens was detected in the sequencing dataset, and no sequence modifications were detected in the obtained full-genome sequence compared with the original cat/SWE/SWE_15/2021 sequence.

### Animals and experimental design

#### Exp. 1: Identification of the route of infection

Five 18-month-old female wood mice (group A), obtained from a private breeder, and five 3-week-old male Lewis rats (group C) (Janvier Labs, Le Genest-Saint-Isle, France) were i.n. inoculated under short-term isoflurane anaesthesia by pipetting 25 µl of the master inoculum equally into both nostrils. The same number of wood mice (group B) and rats (group D) were p.o. inoculated by pipetting 25 µl of the master inoculum into the oral cavity (Table S1, available in the online Supplementary Material). Each group of five animals was housed in one cage. Oral swabs, as well as swabs obtained from the cage walls (environmental swabs), were collected on a weekly basis using dry swabs (Technical Service Consultants Ltd, Heywood, UK) and subsequently stored in 0.5 ml AVL buffer (QIAGEN, Hilden, Germany) before being transferred to −80 °C for long-term storage. Additionally, pooled faecal samples were collected at weekly intervals. At 4 weeks post-infection (wpi), all animals were euthanized, and the following tissues were collected in 1 ml of Minimum Essential Medium and subsequently stored at −80 °C. Tissues were sampled in the following order: spleen, liver, heart, lung, urinary bladder, kidney, adrenal gland, jejunum, colon, parotid gland, nose, eye, cervical spinal cord, lumbar spinal cord, cerebrum and cerebellum. The order of tissue collection was determined based on the viral load observed in prior animal trials to allow harvesting of organs from low to high RNA levels. This procedure was implemented to mitigate potential cross-contamination between organs.

#### Exp. 2: Early stages of infection

A total of seven 22-month-old female wood mice were i.n. inoculated under short-term isoflurane anaesthesia by pipetting 25 µl of the master inoculum equally into both nostrils. Both at 3 (group A)- and 7 (group B)-day post-infection (dpi), two animals were euthanized, while the three remaining animals were euthanized at 14 dpi (group C, Table S2). Oral swabs were collected at days 3, 7 and 14 from all remaining animals, as described above. Tissue samples were harvested as mentioned earlier.

#### Exp. 3: Virus transmission

A total of twelve 18-month-old female wood mice received a combined i.n. and p.o. inoculation (since Exp. 1 had not yet been completed then) under short-term isoflurane anaesthesia by pipetting 25 µl of the master inoculum equally into both nostrils and, in addition, 25 µl into the oral cavity. These directly inoculated donor animals (D1–D12) were kept pairwise (*n*=6 pairs). Six weeks post-inoculation and 3 days following the last change of bedding material, direct contact was initiated by adding one naïve sentinel animal (*n*=6, Sd1–Sd6) to each cage with two donor animals, building six groups in total. In addition, six female wood mice (SiW1–SiW6) and six male Lewis rats (SiLR1–SiLR6) were housed in conspecific pairs (*n*=3 wood mouse pairs and *n*=3 Lewis rat pairs) in the cages previously occupied by the donor animals and the direct contact animal, potentially exposing them to contaminated material (e.g. trough urine or faeces) enabling virus transmission via the environment (Table S3). Cages were changed twice a week from now on, always following the same scheme: each group was moved into clean cages provided with new bedding material. Their used cage was then occupied by an indirect-contact animal pair. Cages were always rotated in the same order: e.g. indirect contact pair 1i always occupied the used cage from donor-direct contact group 1d. Until 6 wpi, sampling was carried out at weekly intervals as previously described for Exp. 1. Thereafter, environmental swabs and pooled faecal samples were collected twice a week. Additionally, rectal swabs of all animals were collected once a week. Necropsy on all animals was performed at 10 wpi, representing a 4-week study period for direct and indirect contact animals. Tissue samples were harvested as described above.

For a detailed overview of the experimental layout, see Fig. S1 with a schematic representation of the experimental setup.

#### Clinical scoring

To assess disease severity, wood mice and juvenile Lewis rats were scored for clinical signs and weighed on a weekly basis. Animals were scored based on their general condition, including the appearance of the fur, posture, behaviour and respiration. Furthermore, animals were scored for trial-specific neurological signs (e.g. ataxia or paresis).

### RNA isolation and detection of viral RNA via reverse transcription-qPCR

Tissue samples collected during necropsy were homogenized with a steel bead using the TissueLyser II (QIAGEN, Hilden, Germany) at an oscillation frequency of 30 Hz for a duration of 3 min. Pooled faecal samples were diluted to a volume of 0.5 to 1.5 ml with PBS, depending on the amount of faeces collected. Oral, rectal and environmental swabs stored in 0.5 ml AVL buffer (QIAGEN, Hilden, Germany) were incubated at 37 °C for 30 min under shaking conditions prior to the isolation of RNA. Nucleic acid extraction was performed using 100 µl of supernatant from tissue and pooled faecal samples and 220 µl from swabs, employing the King Fisher 96 Flex purification system (Thermo Fisher Scientific, Darmstadt, Germany) in combination with the NucleoMag^®^ VET nucleic acid extraction kit (Macherey-Nagel, Düren, Germany), in accordance with the manufacturer’s instructions. Reverse transcription-qPCR reactions were performed as previously described [12]. Subsequent analysis and visualization were conducted using GraphPad Prism 10 and 11.

### Detection of RusV-specific antibodies by an immunofluorescence antibody test

Immunofluorescence antibody test (IFAT) was performed using transfected cells expressing RusV capsid and E2/E1 antigens, as previously described [16]. Starting dilution of the serum was set at 1 : 20. If the sample volume was insufficient, the serum was tested at a dilution of 1 : 40. IFAT was performed in a blinded fashion and evaluated by two separate scientists.

### Histopathology

Tissue collection, processing and light microscopical evaluation were performed as previously described [16]. Briefly, all brain sections were stained with haematoxylin and eosin (H&E). Light microscopy was performed using the digital slide scanning system and ndp.view2 software (version 2.9.29) (both Hamamatsu Photonics, Herrsching, Germany). H&E-stained sections were evaluated and lesions described. Scoring of lesions followed a semiquantitative scheme of four degrees: 1, minimal (<5% of the area is affected or 1–3 foci); 2, mild (6–40% or >3 foci); 3, moderate (41–80% or coalescing); 4, severe (>80% or diffuse). Evaluation and interpretation were performed by a trained pathologist and reviewed by a board-certified pathologist (DiplECVP, AB) in a masked fashion using the post-examination masking method [20].

## Results

### Exp. 1: Identification of the route of infection

In the first experiment (Exp. 1), subgroups of wood mice and juvenile Lewis rats were RusV-inoculated either i.n. or p.o. None of the animals displayed noticeable clinical signs throughout the trial. Body weight of the wood mice remained within the range of physiological fluctuations (Fig. S2A). Throughout the course of the study, juvenile Lewis rats in both experimental groups (i.n. vs. p.o.) exhibited consistent weight gain (Fig. S2B).

**Fig. 1.**
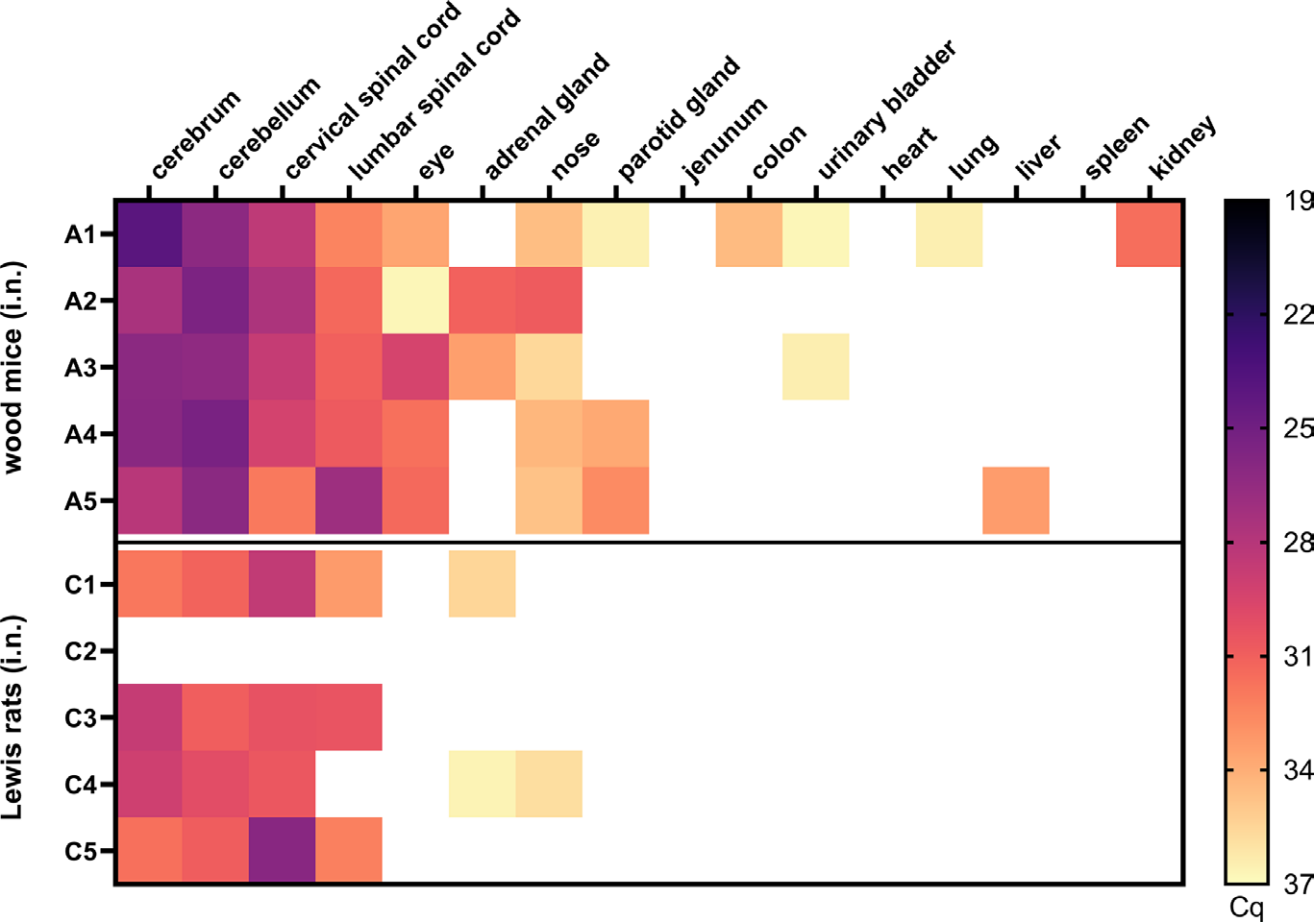
Viral loads determined by RusV-specific reverse transcription-qPCR in tissues of RusV-inoculated wood mice and Lewis rats (Exp. 1). Wood mice and Lewis rats were inoculated intranasally or perorally with RusV-positive material from a previous study. All animals were euthanized 4 wpi. Tissue samples were evaluated for RusV-specific RNA by reverse transcription-qPCR. Colours represent the respective viral load, ranging from black (high load) to light yellow (low load); white squares indicate that viral RNA was not detected. Only intranasally infected wood mice (animals A1–A5) and Lewis rats (animals C1–C5) are shown.

I.n. inoculation resulted in successful infection of all wood mice (A1–A5) and 4 out of 5 Lewis rats (C1, C3–C5), as confirmed by RusV-specific reverse transcription-qPCR (Fig. 1). In contrast, p.o. inoculation did not result in infection of any of the animals (B1–5, D1–5; data not shown). A considerable variation of RusV RNA loads among the sampled organs could be observed. Viral loads ranged from above the detection limit (Cq >37) to levels with Cq values as low as 23.9, as detected in the cerebral tissue of animal A1 (Fig. 1).

The highest viral RNA loads were found in the cerebellum and cerebrum of all i.n. inoculated wood mice (A1–5), with slightly lower loads in the cervical spinal cord, lumbar spinal cord and ocular samples (Fig. 1). Further, we also detected RusV in the adrenal glands of two wood mice (A2, A3). Lower viral loads were present in several mucosal sites: the nose of all animals, the salivary glands of three wood mice (A1, A4, A5), the urinary bladder of two wood mice (A1, A3) and the colon of one mouse (A1) (Fig. 1). Most predominantly parenchymal tissues were negative, with sporadic detection of viral RNA in the kidney, lung and liver.

Analogous to the viral loads detected in i.n. inoculated wood mice, i.n. inoculated Lewis rats showed the highest RusV RNA levels in cerebral and cerebellar tissues, followed by the cervical and lumbar spinal cord tissues of three Lewis rats (C1, C3 and C5). Overall, viral loads in the brains of Lewis rats were lower compared to those of the wood mice (Fig. 1). The adrenal glands of Lewis rats C1 and C4 had low viral loads. Unlike wood mice, eye samples were negative, and mucosal tissues were largely negative except for a single nasal sample (C4). All other tissues tested were RusV-negative.

Only a single oral swab collected from an infected wood mouse (A3) at 4 wpi showed minimal amounts of viral RNA (Cq 36.0, data not shown). All other samples (environmental swabs, pooled faecal samples and rat oral swabs) remained negative for RusV RNA throughout the study. However, it should be noted that a subset of faecal samples could not be evaluated due to inhibitory effects.

**Fig. 2.**
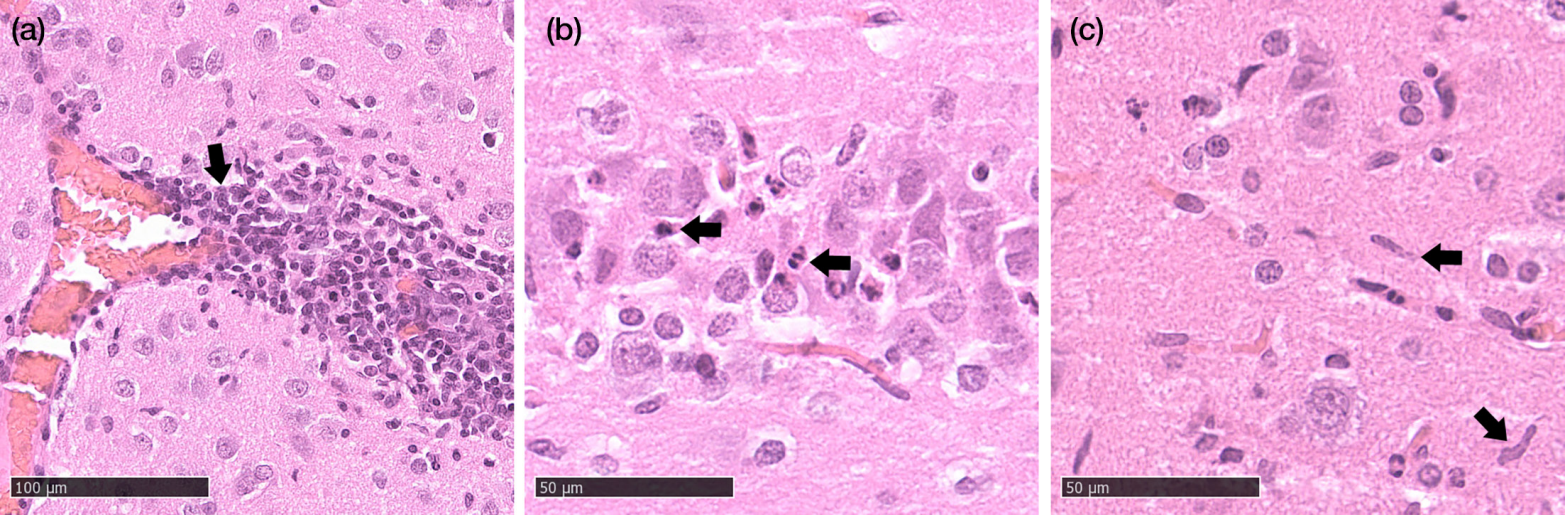
Histopathological lesions in the brains of intranasally inoculated RusV-infected Lewis rats (Exp. 1). (**a**) Perivascular inflammation, (**b**) single cell necrosis/apoptosis and (**c**) microgliosis. Haematoxylin-eosin stain. Bars 100 µm (**a**) and 50 µm (**b, c**).

Histopathological examination by blinded evaluation revealed no lesions in the brains of RusV-infected wood mice after i.n. inoculation (A1–A5). In contrast, minor lesions were observed in all four RusV-positive Lewis rats after i.n. inoculation (C1, C3-5), characterized by perivascular infiltrates and inflammation (1/4), microgliosis (1/4) and minimal to mild single cell necrosis/apoptosis (4/4). Representative lesions are shown in Fig. 2. Rat C2, which was found to be the only uninfected animal after i.n. inoculation, showed no histopathological changes. In line with the reverse transcription-qPCR results, none of the orally inoculated wood mice (B1–B5) and Lewis rats (D1–D5) showed any histopathological findings indicative of RusV infection. The individual raw histopathological scoring data underlying these findings are included in the supplementary material (Table S4).

### Exp. 2: Early stages of infection

In the second experiment (Exp. 2), subgroups of wood mice were inoculated via the i.n. route and euthanized at earlier time points (3, 7 or 14 dpi) to investigate virus invasion. None of the animals included in the second experiment (Exp. 2) displayed noticeable clinical signs throughout the trial. Body weight remained within the range of physiological fluctuations (data not shown).

**Fig. 3.**
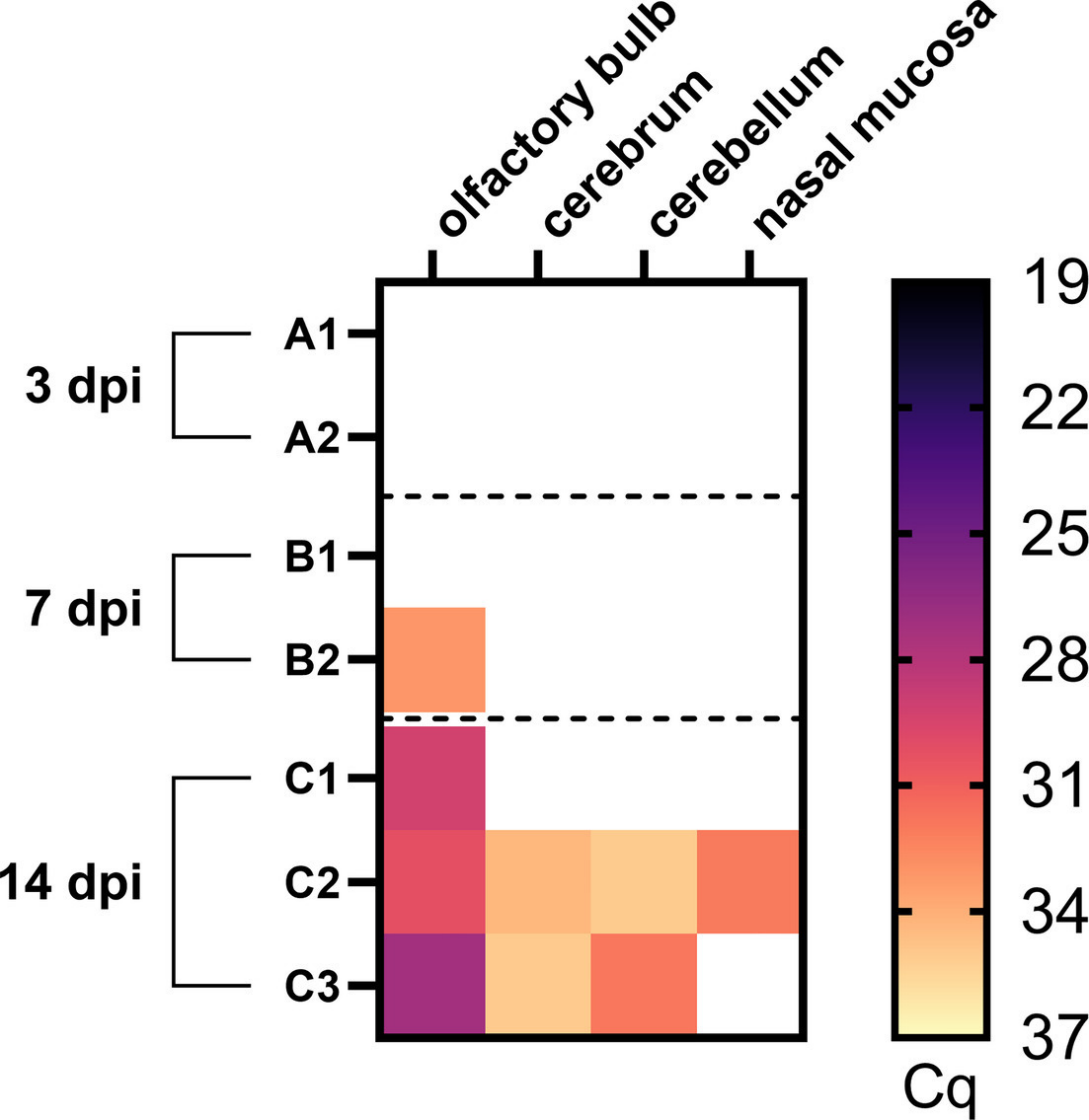
Viral loads determined by RusV-specific reverse transcription-qPCR in tissues of RusV-inoculated wood mice euthanized after 3, 7 and 14 dpi (Exp. 2). In total, seven adult wood mice and juvenile Lewis rats were inoculated intranasally with RusV-positive material from a previous study. At 3 and 7 dpi, two animals (A1 and A2, B1 and B2) each were euthanized, while the three remaining animals were euthanized at 14 dpi (C1–C3). Tissue samples were tested for RusV-specific RNA by reverse transcription-qPCR. Colours represent the respective viral load, ranging from black (high load) to light yellow (low load); white squares indicate that viral RNA was not detected. Only tissue samples that tested positive for RusV are shown.

While reverse transcription-qPCR failed to detect RusV RNA in any organ sampled from animals A1 and A2 (euthanized 3 dpi) or from animal B1 (euthanized 7 dpi), we detected a low amount of RusV RNA in the olfactory bulb of animal B2. Further, viral RNA was detectable in the olfactory bulb of all three animals (C1–C3) euthanized at 14 dpi, with Cq values ranging from 27.2 to 30.1. In addition, we detected viral RNA in the cerebrum (Cq >34.0) and cerebellum (Cq 31.9 and 35.0) of animals C2 and C3. Detection of RusV-specific RNA in nasal tissue was only observed in animal C2 (Cq >34.0) (Fig. 3).

All other tissue samples and oral swabs were negative for RusV (data not shown).

### Exp 3: Virus transmission

In Exp. 3, we investigated whether RusV can be transmitted from RusV-inoculated donor animals to sentinel animals via direct or indirect contact. None of the wood mice (D1–D12, Sd1–Sd6 and SiW1–SiW6) included in Exp. 3 showed clinical signs. Body weight remained within the range of physiological fluctuations (Fig. S3A). Juvenile Lewis rats (SiLR1–SiLR6) displayed consistent weight gain (Fig. S3B).

**Fig. 4.**
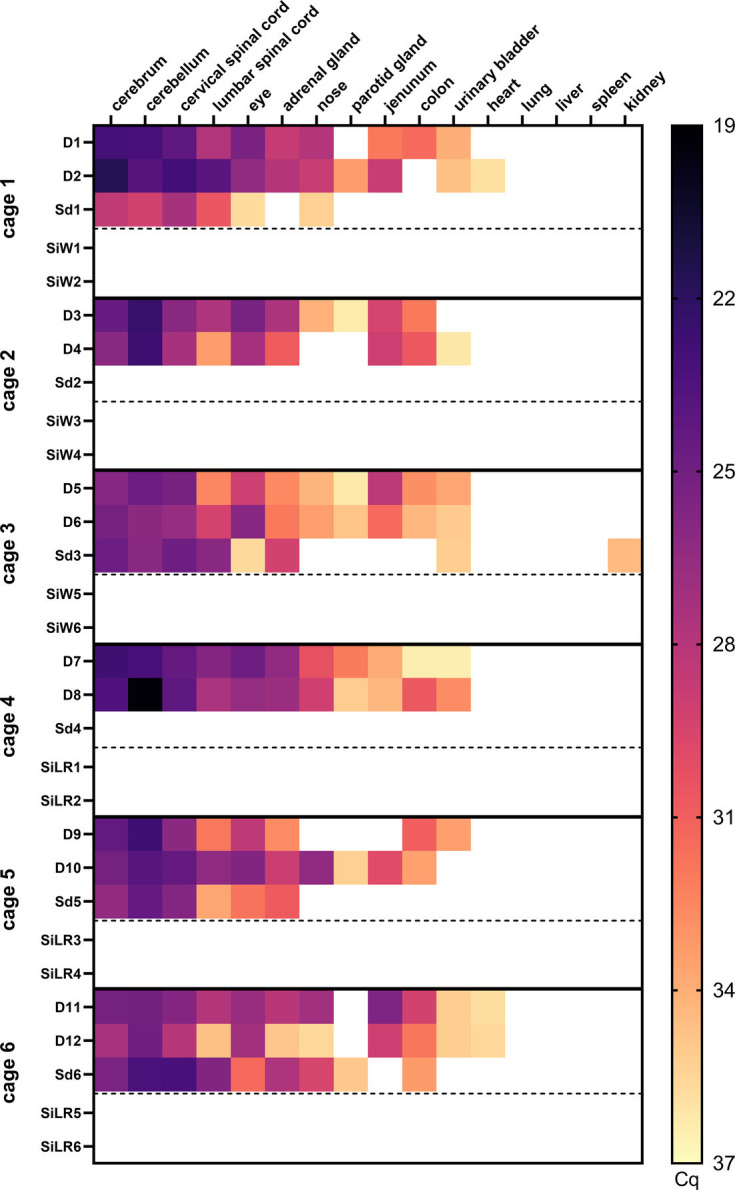
Viral loads determined by RusV-specific reverse transcription-qPCR in tissues of RusV-inoculated donor animals and direct and indirect contact animals (Exp. 3). Adult wood mice (donor animals D1–D12) were inoculated intranasally and perorally with RusV. After a period of 6 weeks, non-inoculated sentinel animals (Sd1–Sd6) were co-housed with donor animals (D1–D12). At 10 wpi, all donor animals (D1–D12) were euthanized, and 4 weeks after the initiation of direct contact, all sentinel animals (Sd1–Sd6) were euthanized, and tissue samples were harvested. Tissue samples were tested for RusV-specific RNA by reverse transcription-qPCR. Colours represent the respective viral RNA load, ranging from black (high load) to light yellow (low load); white squares indicate that viral RNA was not detected. Dashed lines indicate that SiW1-6 and SiLR1-6 were transferred to the contaminated cages previously housing D1–D12 and Sd1–Sd6 (see Fig. S1 and Table S3 for detailed experimental setup). D, donor; Sd, sentinel direct; Si, sentinel indirect.

RusV-specific reverse transcription-qPCR confirmed infection in all donor animals (D1–D12) euthanized at 10 wpi and 4 out of 6 direct contact animals (Sd1, Sd3, Sd5–Sd6) euthanized 4 weeks after co-housing. In contrast, all indirect contact wood mice (SiW1–SiW6) and Lewis rats (SiLR1–SiLR6) remained negative for RusV RNA (Fig. 4).

Consistent with our results from Exp. 1 and 2, we detected viral RNA predominantly in neuronal tissue of the successfully infected donor animals (Fig. 4). Highest viral loads were found in cerebral and cerebellar tissues. This was followed by the cervical and lumbar parts of the spinal cord. Moreover, viral RNA was found in the eye and in the adrenal gland. Viral RNA was also detected in mucosal tissues, such as the nose (10/12 animals), salivary gland (7/12 animals) and urinary bladder (10/12 animals). Notably, viral RNA was also detected in the jejunum (except for animal D9). A similar pattern was observed for colon samples (11/12 animals). Low viral loads were detected in the heart (animals D2, D11 and D12) (Fig. 4).

The distribution of viral RNA detected in contact animals Sd1, Sd3, Sd5–Sd6 follows a similar pattern as described for D1–D12, with the highest RusV RNA loads detected in cerebral and cerebellar tissues, followed by the cervical and lumbar spinal cord. In addition, slightly lower viral loads were detected in ocular samples and adrenal glands (except for animal Sd1). Mucosal tissue, such as nasal tissue, was positive in two of the successfully infected direct contact animals (Sd1 and Sd6). However, in contrast to the donor animals, viral RNA was not detected in jejunum samples, and only single animals had detectable amounts of viral RNA in the salivary gland (Sd6), urinary bladder (Sd3), kidney (Sd3) and colon (Sd6).

**Fig. 5.**
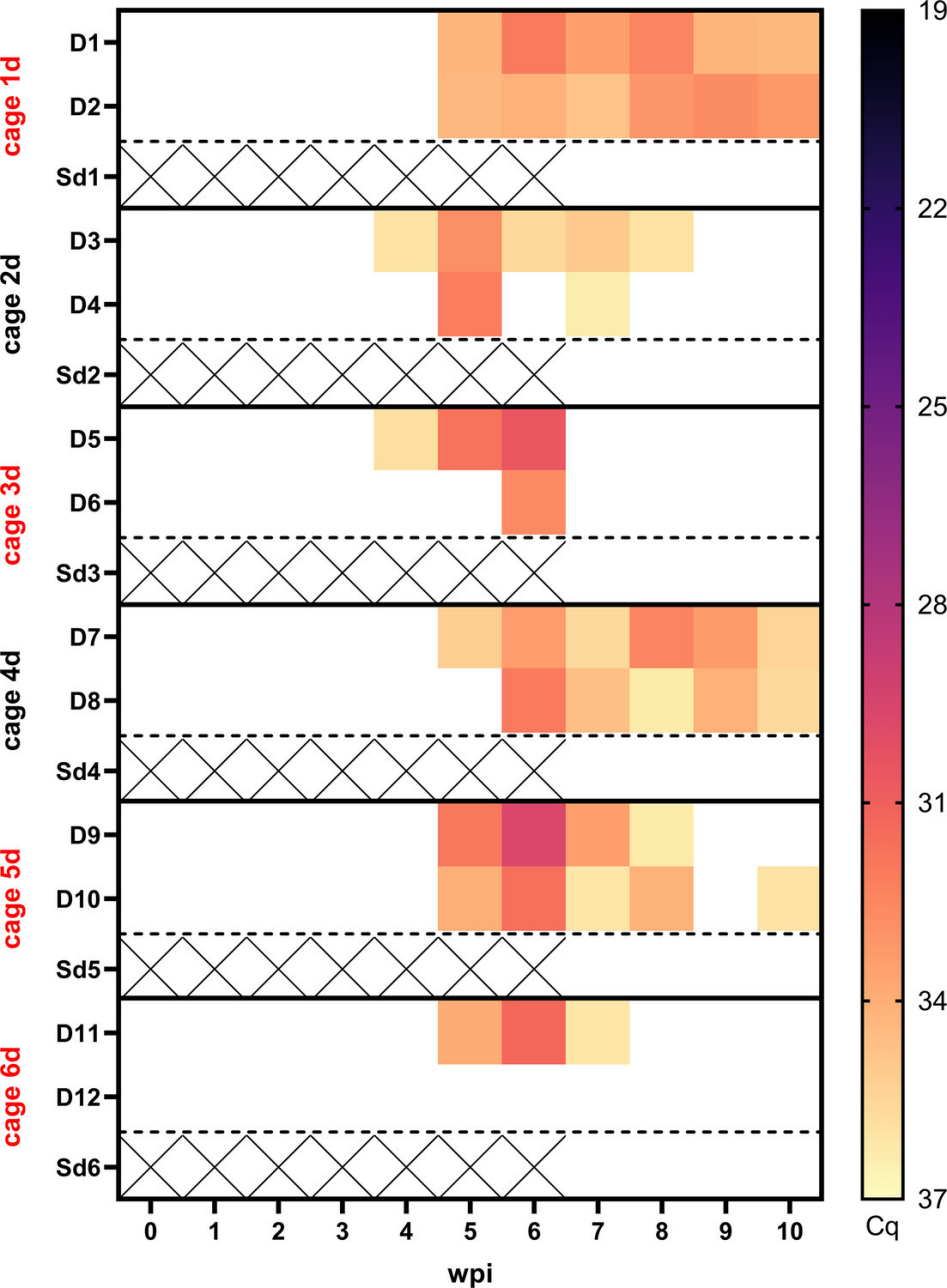
Viral RNA detected in sampled oral swabs of RusV-inoculated donor animals (Exp. 3). Adult wood mice (donor animals D1–D12) were inoculated intranasally and perorally with RusV-positive material from a previous study. After a period of 6 weeks, non-inoculated sentinel animals Sd1–Sd6 were co-housed with donor animals D1–D12 (see Fig. S1 and Table S3 for detailed experimental setup). During the study, oral swabs of all animals were collected at weekly intervals. All samples were evaluated for RusV-specific RNA by reverse transcription-qPCR. Squares are coloured according to the respective viral RNA load, ranging from black (high load) to light yellow (low load); white squares indicate that viral RNA was not detected; black crosses indicate that no oral swab samples were collected for direct contact animals during weeks 0–5, as these animals were introduced into the experiment 6 weeks after inoculation of the donor animals. Cage numbers shown in red indicate successful viral transmission, as determined by RusV-specific reverse transcription-qPCR-positive animals. d, direct; D, donor.

RNA extracted from oral swabs confirmed the presence of RusV-specific RNA in at least one sample from 11 out of 12 donor animals, except for animal D12 (Fig. 5). RusV RNA in the oral cavity was detected in animals D3 and D5 as early as 4 wpi. From 5 to 7 wpi, viral RNA was detected in 9 to 10 out of 12 donor animals. In week 8, we detected RusV RNA in only 7 out of 12 donor animals, with slightly lower RNA levels. This was followed by the detection of viral RNA in 4 out of 12 donor animals in week 9 and 5 out of 12 donor animals in week 10. Furthermore, viral shedding could not be detected in the successfully infected direct contact animals (Fig. 5).

Sporadically, low levels of viral RNA were detected in environmental swabs taken from the cage walls and bedding material. In contrast, viral RNA was not detected in rectal swabs or pooled faecal samples from either the successfully infected donor or direct contact animals (data not shown). However, it should be noted that a subset of faecal samples was not suitable for molecular analysis due to inhibitory effects.

RusV-reactive antibodies were not detectable by IFAT in any of the sera collected at euthanasia throughout all three experiments (data not shown).

**Fig. 6.**
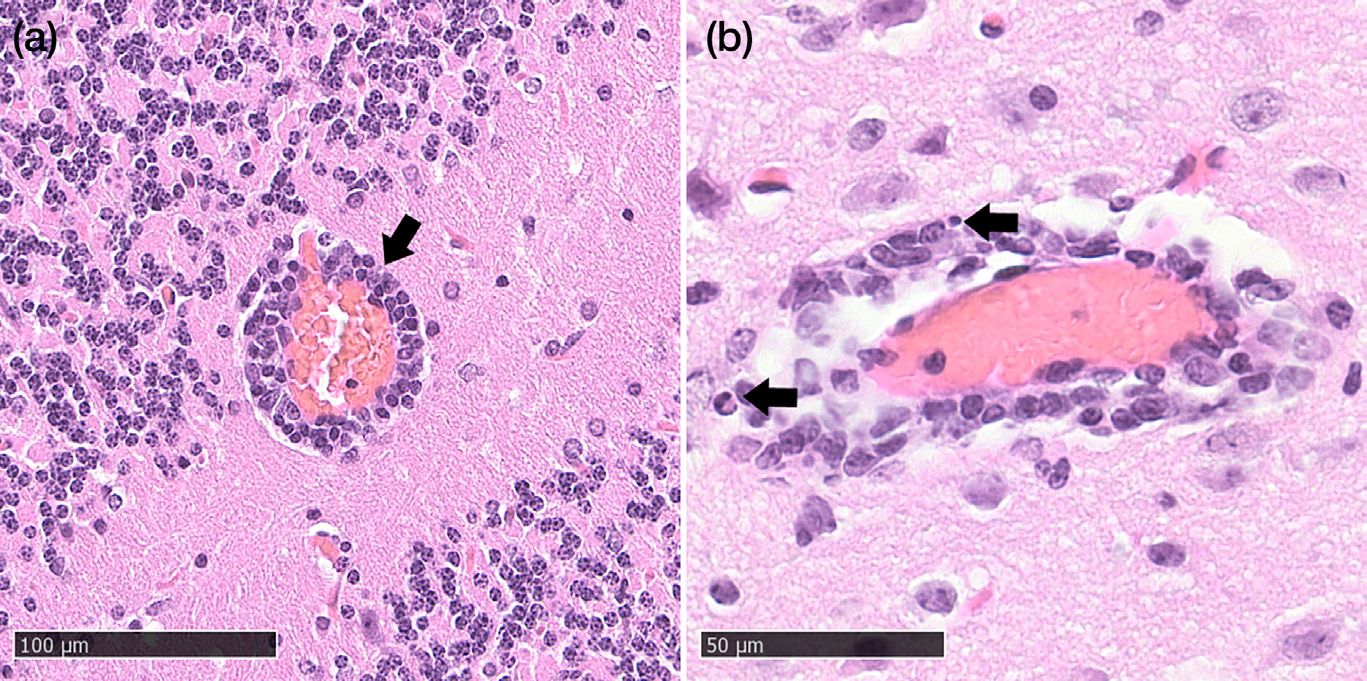
Histopathological lesions in the brains of intranasally inoculated RusV-infected wood mice (donor animals). (**a**) perivascular lymphohistiocytic infiltrates and (**b**) perivascular inflammation. Haematoxylin–eosin stain. Bars 100 µm (**a**) and 50 µm (**b**).

Histopathology of donor animals (D1–D12) revealed minor lesions in the brain of 8 out of 12 inoculated and successfully infected mice, showing perivascular lymphohistiocytic infiltrates (‘perivascular cuffing’, 8/12), partially associated with the activation of the vascular endothelium or cell death within the perivascular space (‘inflammation’, 6/12), microgliosis (3/12) and single cell necrosis/apoptosis (2/12). One infected, direct contact mouse (Sd6) exhibited minimal perivascular inflammation, and one non-infected indirect contact mouse (SiW3) showed minimal perivascular infiltrates. Representative lesions are shown in Fig. 6. Individual histopathological scores for all examined animals are included in the supplementary material (Table S5).

## Discussion

After the primary infection route of RusV could be narrowed down to at least mucosal inoculation [16], we were able to successfully uncover the primary route of infection with RusV to be intranasal, as only i.n. inoculated wood mice and Lewis rats, but none of the p.o. inoculated animals were RusV-positive, as confirmed by reverse transcription-qPCR (Fig. 1). Interestingly, our results from Exp. 2 demonstrate that viral RNA is first detected in the olfactory bulb at 7 dpi (1/2 animals), followed by the detection of RusV RNA in the olfactory bulb of all three animals euthanized at 14 dpi. In the cerebrum and cerebellum, viral RNA was first detected at 14 dpi (2/3 animals; Cq >31) (Fig. 3). In contrast to the neurotropic rabies virus, viral RNA is detected at a substantially later stage in the olfactory bulb [21]. Conversely, for the neurotropic Borna disease virus 1, viral RNA was detected in the olfactory bulb at 6 dpi, which aligns more closely with our findings [22]. The underlying mechanisms for the rather late detection of RusV RNA in the olfactory bulb remain unclear. Hypothetically, it is plausible that the virus is retained or neutralized during its passage through the mucus layers that coat the olfactory epithelium, which may restrict or delay subsequent entry into the olfactory bulb. This mucosal barrier may act as a physical and immunological filter that temporarily traps viral particles and thereby delays neuroinvasion despite early exposure at the epithelial surface [23]. On the other hand, it could also be that RusV replicates very slowly and at a low level and thus happens to be absent from the sampled sections at day 3.

Our results from Exp. 1 and 2 strongly suggest that viral invasion and spread are initiated in the nasal epithelium, subsequently disseminating to the olfactory bulb and ultimately reaching the entire CNS. This mechanism of transolfactory neuroinvasion has been demonstrated by experimental i.n. inoculation for many other neurotropic viruses, including polio and rabies virus, but also for respiratory viruses with manifestation in the CNS such as the PR8 strain of influenza A virus [24,27]. However, the specifics of the invasion pattern of RusV remain to be determined. Further studies should, therefore, focus on the olfactory neuroepithelium, including sensory nerves, to investigate initial replication sites as well as neuroinvasion.

In accordance with the previous results of Klein *et al*. [16], we consistently observed the highest viral loads in brain and spinal cord tissues, as indicated by the lowest Cq values detected in these tissues throughout all three experiments. This finding is further consistent with previous reports from naturally RusV-infected animals [3,8, 9, 12, 16, 18]. Moreover, in Exp. 1, viral RNA levels in the brains of wood mice euthanized at 28 dpi exceeded those in the brains of Lewis rats (a difference of four Cq values). Similar differences in viral RNA levels in brain tissue of these species were reported in the study of Klein *et al*. [16]. These findings suggest that RusV may exhibit enhanced replicative fitness and adaptability in its natural reservoir (wood mouse), whereas it differs from dead-end hosts (Lewis rats), in which viral replication may be more limited.

Outside the CNS, viral RNA was also present at lower levels in a variety of non-CNS organs, but its tissue distribution pattern varied between the different experiments and between both species. The distributional divergence of viral RNA between wood mice and Lewis rats in Exp. 1 regarding organs with a high proportion of mucosal tissue was notable, as viral RNA was detected more frequently in these organs in wood mice compared to Lewis rats (Fig. 1). In Exp. 3, the spread of viral RNA in organs with a high proportion of mucosal tissue was remarkable (Fig. 4). Interestingly, we detected a less pronounced spread of viral RNA in organs with a high proportion of mucosal tissue in the infected direct contact animals compared to the donor animals. This was especially noticeable in the following organs: salivary gland, jejunum, colon and urinary bladder (Fig. 4). Here, it should be noted that donor animals were euthanized at 10 wpi, whereas direct contact animals had been exposed to them for only 4 weeks. Moreover, the exact timing of infection in the direct contact animals during the exposure period remains unknown. In further sampled organs such as the heart, lungs, liver, spleen and kidneys, viral RNA was consistently undetectable or present at only very low levels across all three experiments. These results corroborate reports of naturally and experimentally infected animals [3,12, 16].

Consistent with previously published data, reporting detection of viral RNA in oral swabs of experimentally infected wood mice and naturally infected yellow-necked field mice [3,16], we likewise detected RusV RNA in oral swabs sampled from 11 out of 12 donor animals as early as 4 wpi with moderate to low viral loads (Fig. 5). Further, in our study rectal swabs and pooled faecal samples were negative for viral RNA (data not shown), mirroring a prior study that detected only low amounts of viral RNA in a limited number of faecal samples collected from wood mice [16]. However, some of the stool samples were not suitable for molecular analysis due to limitations in sample quality, as reverse transcription-qPCR inhibition was suspected. Environmental swabs collected from cage walls and contaminated bedding material revealed infrequent detection of viral RNA, with only a small subset of swabs testing positive, albeit at very low RNA levels (Cq >34) (data not shown).

Although viral RNA has been consistently detected in oral swabs, implying a potential route of transmission via saliva, recent RNA-ISH analyses revealed the virus only within neuronal components of mucosal tissues and failed to identify it in epithelial cells of infected wood mice [16]. Nevertheless, when taking the oral swab, it is possible that not only saliva but also cells from the oral mucosa are sampled. Demonstrating the infectivity of saliva through virus isolation from oral swabs would further support this hypothesis; however, a suitable *in vitro* system is still missing. Detected viral RNA in environmental swabs may originate from saliva, consistent with viral RNA detected in oral swabs (Fig. 5), although other potential routes of shedding, such as urine, which were not investigated in this study, cannot be ruled out. Whether RusV is in fact shed via additional routes like lacrimal fluid or urine, as observed for the highly neurotropic Borna disease virus 1 in infected bicoloured white-toothed shrews [28], should be addressed in follow-up studies.

In Exp. 3, we successfully confirmed RusV-infection in 4 out of 6 of direct contact animals. In contrast, we failed to demonstrate transmission of virus to indirect contact animals, as evidenced by the absence of RusV RNA in all the tissues sampled (Fig. 4). Therefore, our findings suggest that any excretion of infected donor animals did not result in infection of indirectly exposed animals.

Combined, these findings suggest two possible interpretations: (i) RusV exhibits limited environmental persistence, thereby reducing the events of indirect transmission, and/or (ii) the infectious dose required to establish infection in animals without direct contact to infected reservoirs exceeds the amount present in the contaminated environment.

In summary, our data suggest that direct contact with an infected wood mouse or yellow-necked field mouse is required for infection, whereas indirect contact by exposure to a contaminated environment is apparently not sufficient for viral transmission.

Direct contact with infected reservoir hosts is a plausible mode of transmission, particularly for outdoor cats and other wild carnivores that actively hunt and prey upon small mammals, thereby increasing their probability of encountering infected reservoirs. In contrast, certain zoo animals, such as capybaras and red-necked wallabies, which have been previously reported to be infected by RusV, do not typically engage in predatory behaviour towards small mammals, which makes direct contact with reservoir animals less likely [3,9]. Nevertheless, the possibility of a direct encounter between these animals and infected reservoirs, particularly in areas where they gather for feeding or resting, cannot be excluded, especially with regard to infected reservoirs found to be present within the zoo premises [3].

In a previous study by Klein *et al*., only low levels of RusV-specific antibodies were detected at 8 to 12 wpi in a subset of wood mice inoculated via the mucosal route, whereas i.c. inoculated wood mice, as well as Lewis rats inoculated via i.c. or mucosal routes did not exhibit seroconversion [16]. In this study, we did not observe any seroconversion in infected wood mice for up to 10 wpi (Exp. 3). These results suggest two explanations: (i) the sensitivity of the established IFAT may be limited, or (ii) seroconversion following RusV infection is a rare event. To date, no alternative serological assays for detecting RusV-specific antibodies have been reported.

Histopathological examination confirms once again that infection with RusV leads to non-suppurative inflammation in the CNS, which has been identified in both Lewis rats and wood mice. In the study by Klein *et al*., experimentally infected wood mice were affected less consistently and to a lesser degree than Lewis rats [16]. However, the evidence of an inflammatory response after infection can also occur in reservoir hosts and is, therefore, not surprising.

## Conclusions

This study deciphered the primary route of infection with RusV to be intranasal. Further, our study demonstrated that only direct contact with infected reservoir hosts resulted in infection of sentinel animals, but not indirect contact. Therefore, this study provides the basis for future research to investigate the mechanisms of RusV neuroinvasion.

## Supplementary material

10.1099/jgv.0.002266 Uncited Supplementary Material 1.

## Abbreviations

CNS: central nervous system
Cq: cycle of quantification
dpi: days post-infection
H&E: haematoxylin and eosin
IFAT: immunofluorescence antibody test
i.n.: intranasal
p.o.: peroral
qPCR: quantitative PCR
RNA-ISH: RNA *in situ* hybridization
RusV: rustrela virus
RuV: rubella virus
wpi: weeks post-infection
