# Supplementary material for: Further characterization of infection mechanisms and transmission dynamics of rustrela virus in rodent in vivo models
Source: J Gen Virol. 2026 May 19;107(5):002266. doi: 10.1099/jgv.0.002266 (PMC13186285; doi:10.1099/jgv.0.002266)
Supplement: Uncited Supplementary Material 1. [file jgv-107-02266-s001.pdf]

Supplementary material

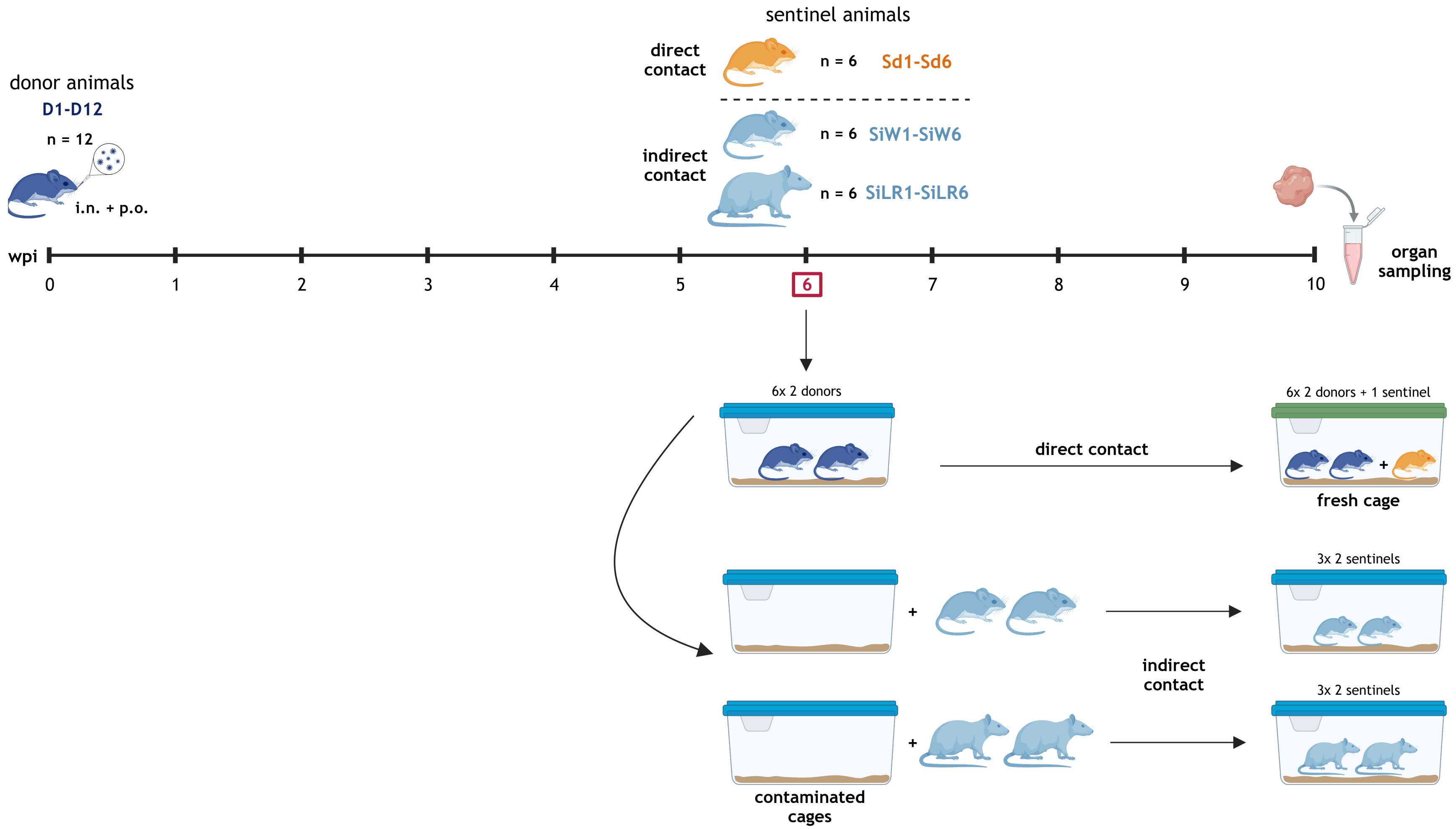

Supplementary Figure S1. Twelve 18-month-old female wood mice (D1–D12) were inoculated intranasally (i.n.) and perorally (p.o.) with a RusV inoculum and housed in pairs. After 6 weeks, each cage received one naïve wood mouse sentinel (Sd1–Sd6) for direct contact. Six additional wood mouse sentinels (SiW1–SiW6) and six Lewis rat sentinels (SiLR1–SiLR6) were later placed in the same cages, species-specific, to assess indirect exposure; cages were switched twice weekly. Donors were euthanized at 10 weeks post-infection (wpi); all sentinels were euthanized 4 weeks after initiation of the direct and indirect contact phase and a full organ panel was collected. Icons: dark blue = donors (D); orange = sentinels direct (Sd); light blue = sentinels indirect - wood mice (SiW1-SiW6) and sentinels indirect – Lewis rats (SiLR1-SiLR6).

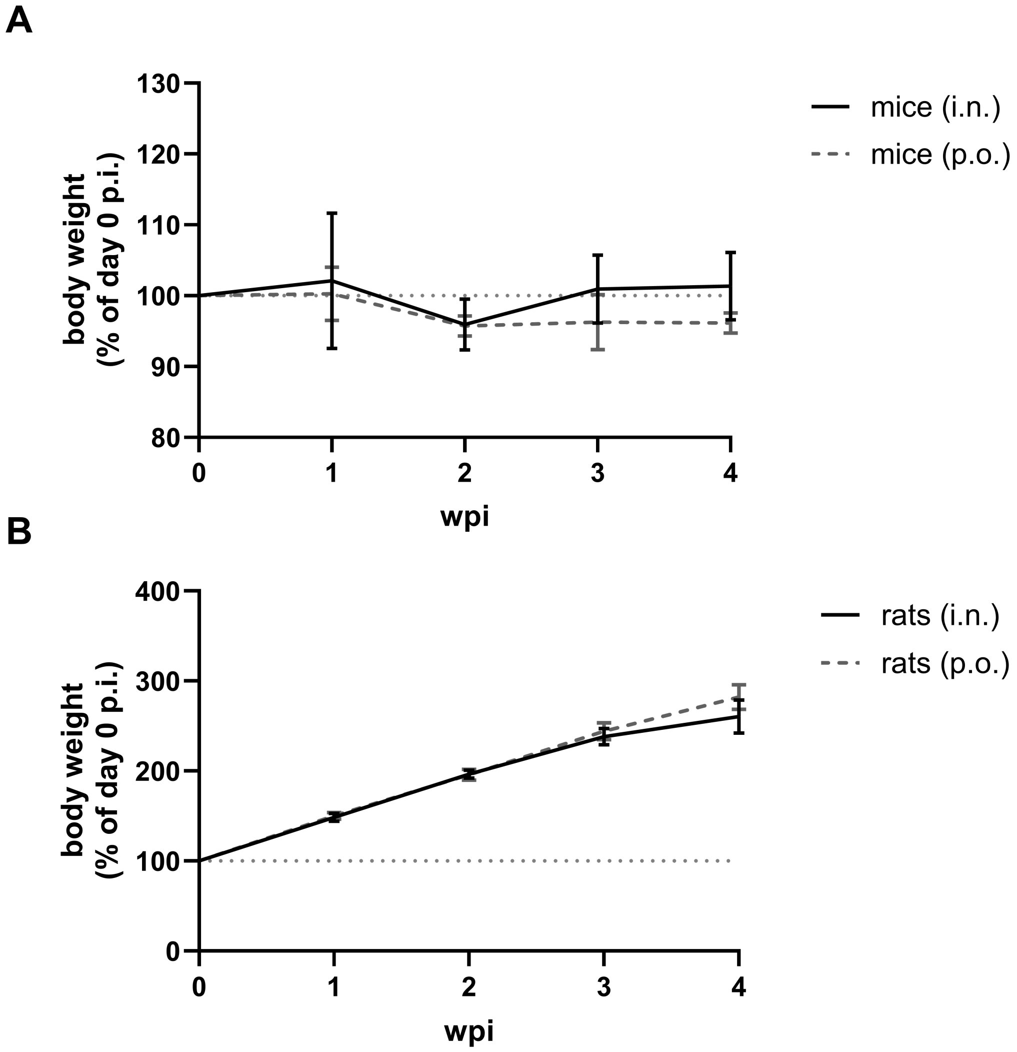

Supplemental Figure S2. Body weight of RusV-inoculated wood mice and Lewis rats (Exp. 1). Adult wood mice (A) and juvenile Lewis rats (B) were inoculated intranasally (i.n.; solid black lines) or perorally (p.o.; dashed gray lines). Body weights were determined at weekly intervals. Results are presented as arithmetic means (+/- standard deviation) per group of the relative body weight (% of the body weight at the start of the experiment; day 0 post-infection). p.i., post-infection; wpi, week post-infection.

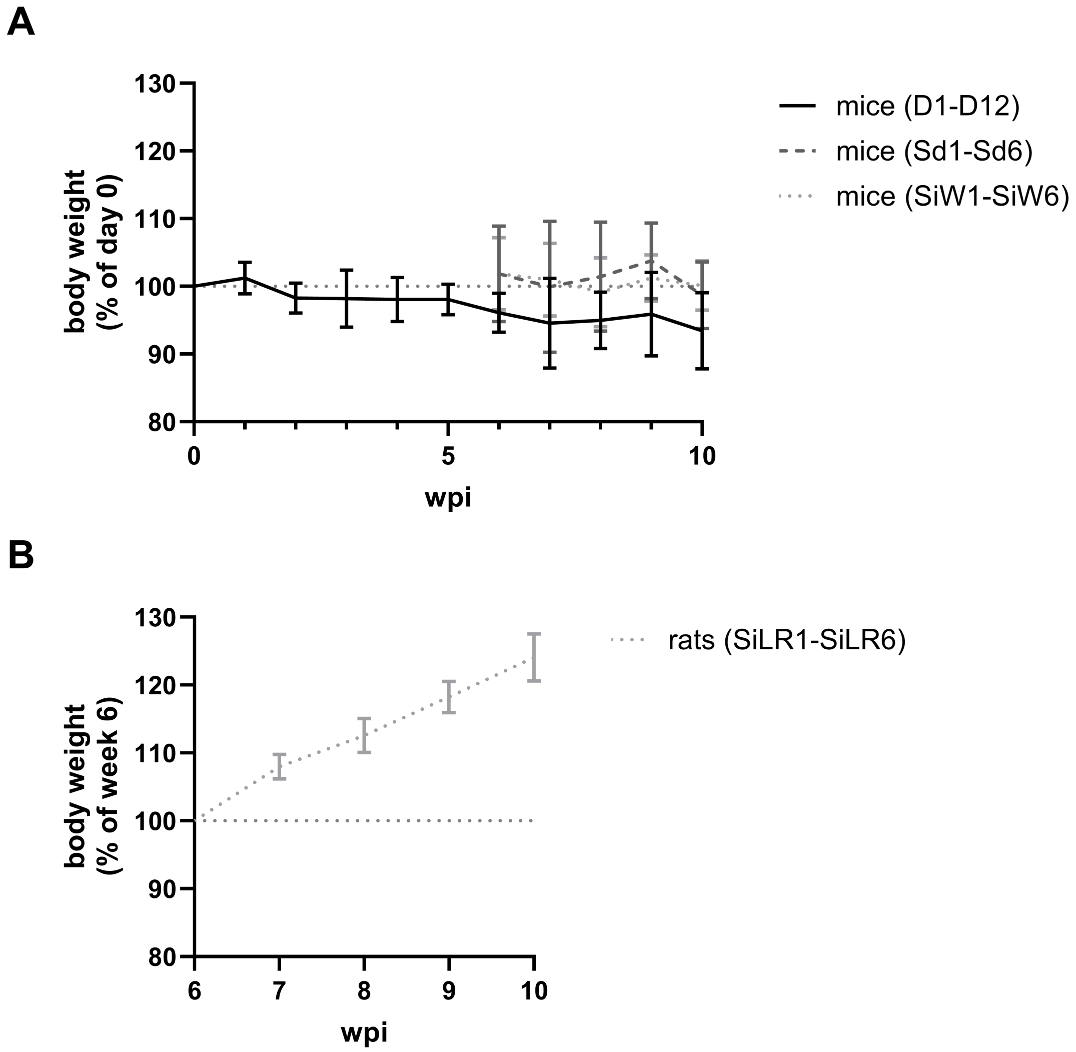

Supplementary Figure S3. Body weight of wood mice (A) and Lewis rats (B) (Exp. 3). Donor wood mice (D1-D12) (A) (solid black line) were inoculated intranasally (i.n.) and perorally (p.o.) with RusV. The dashed gray line represents direct contact wood mice (Sd1-Sd6) (A) and the dotted gray line represents indirect contact animals (SiW1-SiW6 and SiLR1-SiLR6 in (A) and (B), respectively). Results are presented as arithmetic means (+/- standard deviation) per group of the relative body weight (% of the body weight at the start of the experiment; day 0 post-infection. wpi, week post-infection.

Supplementary Table S1. Experimental design of Exp. 1.

Group	Species	Inoculation route [a]	No. of animals	Euthanasia at dpi [b]
A (A1-5)	wood mice	i.n.	5	28
B (B1-5)		p.o.	5	
C (C1-5)	Lewis rats	i.n.	5	
D (D1-5)		p.o.	5	

[a] i.n., intranasal; p.o., peroral

[b] dpi, days post-infection

Supplementary Table S2. Experimental design of Exp. 2.

Group	Species	Inoculation route [a]	No. of inoculated animals	Euthanasia at dpi [b]
A (A1-2)	wood mice	i.n.	2	3
B (B1-2)			2	7
C (C1-3)			3	14

[a] i.n., intranasal

[b] dpi, days post-infection

Supplementary Table S3. Experimental design of Exp. 3.

Group [a]	Species	Role	Inoculation route [d]	No. of animals	Euthanasia at day post-infection / start of contact period
D (D1-D12)	wood mice	RusV-inoculated donor animals	i.n./p.o.	12	70
Sd (Sd1-Sd6)		Sentinel animals with direct contact to donor animals (i.e. direct contact animals) [b]	-	6	28
SiW (SiW1-SiW6)	wood mice	Sentinel animals with indirect contact to donor animals (i.e. indirect contact animals) [c]		6	
SiLR (SiLR1-SiLR6)	Lewis rats			6	

[a] D, donor; Sd, sentinel direct; Si, sentinel indirect

[b] contact to donor animals was initiated 6 weeks after inoculation of D1-D12

[c] first exposure to contaminated cages occurred 6 weeks after inoculation of D1-D12

[d] i.n./p.o., intranasal/peroral

Supplemental Table S4 **Exp. 1: Identification of the route of infection**

	group:	A					B					C					D				
	animal ID:	A1	A2	A3	A4	A5	B1	B2	B3	B4	B5	C1	C2	C3	C4	C5	D1	D2	D3	D4	D5
		wood mice; i.n.					wood mice; p.o.					Lewis rats; i.n.					Lewis rats; p.o.				
	RusV RT-qPCR status:	pos	pos	pos	pos	pos	neg	neg	neg	neg	neg	pos	neg	pos	pos	pos	neg	neg	neg	neg	neg
	cumulative score (brain/spinal cord):*	**0**	**0**	**0**	**0**	**0**	**0**	**0**	**0**	**0**	**0**	**2**	**0**	**2**	**2**	**5**	**0**	**0**	**0**	**0**	**0**
**tissue**	**finding**																				
**brain**	pv inflammation	0	0	0	0	0	0	0	0	0	0	0	0	0	0	1	0	0	0	0	0
	pv infiltrates / cuffing	0	0	0	0	0	0	0	0	0	0	0	0	0	0	1	0	0	0	0	0
	microgliosis	0	0	0	0	0	0	0	0	0	0	0	0	0	0	1	0	0	0	0	0
	single cell necrosis / apoptosis	0	0	0	0	0	0	0	0	0	0	2	0	1	2	2	0	0	0	0	0
	neuronal necrosis, hippocampus	0	0	0	0	0	0	0	0	0	0	2	0	1	2	2	0	0	0	0	0
**spinal cord**	pv inflammation	0	0	0	0	0	0	0	0	0	0	0	0	0	0	0	0	0	0	0	0
	pv infiltrates / cuffing	0	0	0	0	0	0	0	0	0	0	0	0	1	0	0	0	0	0	0	0
	microgliosis	0	0	0	0	0	0	0	0	0	0	0	0	0	0	0	0	0	0	0	0
	single cell necrosis / apoptosis	0	0	0	0	0	0	0	0	0	0	0	0	0	0	0	0	0	0	0	0
**nose**	no finding	0	0	0	0	0	0	0	0	0	0	0	0	0	0	0	0	0	0	0	0

Lesion score

0	No lesion
1	Rare (<5%), 1-3 foci
2	Multifocal (6–40%) >3 foci
3	Coalescing (41–80%)
4	Diffuse (>80%)

i.n. = intranasal
p.o. = peroral

*The single cell necrosis / apoptosis score refers to the entire brain, including the hippocampus. The hippocampal necrosis score therefore represents an informative subscore rather than an independent parameter and is not included in the cumulative score. Consequently, animals that exhibit necrosis in the hippocampus also show necrosis in the brain overall.

Supplemental Table S5 **Exp. 3: Virus transmission**

tissue	finding	D (D1-D12) / wood mice											
	animal ID:	D1	D2	D3	D4	D5	D6	D7	D8	D9	D10	D11	D12
	role:	RusV-inoculated donor animals; i.n. + p.o.											
	RusV RT-qPCR status:	pos	pos	pos	pos	pos	pos	pos	pos	pos	pos	pos	pos
	cumulative score (brain/spinal cord):*	**0**	**2**	**4**	**4**	**1**	**0**	**0**	**0**	**6**	**1**	**2**	**5**
**tissue**	**finding**												
**brain**	pv inflammation	0	1	1	1	0	0	0	0	1	0	1	2
	pv infiltrates / cuffing	0	1	2	2	1	0	0	0	2	1	1	2
	microgliosis	0	0	1	0	0	0	0	0	1	0	0	1
	single cell necrosis / apoptosis	0	0	0	1	0	0	0	0	1	0	0	0
	neuronal necrosis, hippocampus	0	0	0	0	0	0	0	0	0	0	0	0
**spinal cord**	pv inflammation	0	0	0	0	0	0	0	0	0	0	0	0
	pv infiltrates / cuffing	0	0	0	0	0	0	0	0	1	0	0	0
	microgliosis	0	0	0	0	0	0	0	0	0	0	0	0
	single cell necrosis / apoptosis	0	0	0	0	0	0	0	0	0	0	0	0
**nose**	no finding	0	0	0	0	0	0	0	0	0	0	0	0

tissue	finding	Sd (Sd1-Sd6) / wood mice						SiW (SiW1-SiW6) / wood mice						SiLR (SiLR1-SiLR6) / Lewis rats					
	animal ID:	Sd1	Sd2	Sd3	Sd4	Sd5	Sd6	SiW1	SiW2	SiW3	SiW4	SiW5	SiW6	SiLR1	SiLR2	SiLR3	SiLR4	SiLR5	SiLR6
	role:	direct contact animals; not inoculated						indirect contact animals; not inoculated						indirect contact animals; not inoculated					
	RusV RT-qPCR status:	pos	neg	pos	neg	pos	pos	neg	neg	neg	neg	neg	neg	neg	neg	neg	neg	neg	neg
	cumulative score (brain/spinal cord):*	**0**	**0**	**0**	**0**	**0**	**2**	**0**	**0**	**1**	**0**	**0**	**0**	**0**	**0**	**0**	**0**	**0**	**0**
**tissue**	**finding**																		
**brain**	pv inflammation	0	0	0	0	0	1	0	0	0	0	0	0	0	0	0	0	0	0
	pv infiltrates / cuffing	0	0	0	0	0	1	0	0	1	0	0	0	0	0	0	0	0	0
	microgliosis	0	0	0	0	0	0	0	0	0	0	0	0	0	0	0	0	0	0
	single cell necrosis / apoptosis	0	0	0	0	0	0	0	0	0	0	0	0	0	0	0	0	0	0
	neuronal necrosis, hippocampus	0	0	0	0	0	0	0	0	0	0	0	0	0	0	0	0	0	0
**spinal cord**	pv inflammation	0	0	0	0	0	0	0	0	0	0	0	0	0	0	0	0	0	0
	pv infiltrates / cuffing	0	0	0	0	0	0	0	0	0	0	0	0	0	0	0	0	0	0
	microgliosis	0	0	0	0	0	0	0	0	0	0	0	0	0	0	0	0	0	0
	single cell necrosis / apoptosis	0	0	0	0	0	0	0	0	0	0	0	0	0	0	0	0	0	0
**nose**	no finding	0	0	0	0	0	0	0	0	0	0	0	0	0	0	0	0	0	0

Lesion score

0	No lesion
1	Rare (<5%), 1-3 foci
2	Multifocal (6–40%) >3 foci
3	Coalescing (41–80%)
4	Diffuse (>80%)

i.n. = intranasal
p.o. = peroral

*The single cell necrosis / apoptosis score refers to the entire brain, including the hippocampus. The hippocampal necrosis score therefore represents an informative subscore rather than an independent parameter and is not included in the cumulative score. Consequently, animals that exhibit necrosis in the hippocampus also show necrosis in the brain overall.
